# Sustained Treatment Success With Ustekinumab in Symptomatic Stricturing Crohn's Disease: A Retrospective Single‐Arm Observational Cohort Study

**DOI:** 10.1002/mco2.70824

**Published:** 2026-06-17

**Authors:** Jingwen Liu, Wen Hu, Shuyan Li, Shurong Hu, Deming Jiang, Yan Chen

**Affiliations:** ^1^ Department of Gastroenterology, The Second Affiliated Hospital Zhejiang University School of Medicine Hangzhou China; ^2^ Department of Gastroenterology The First Affiliated Hospital of Zhejiang Chinese Medical University (Zhejiang Provincial Hospital of Chinese Medicine) Hangzhou China; ^3^ Department of Nursing, The Second Affiliated Hospital Zhejiang University School of Medicine Hangzhou China; ^4^ Laboratory Animal Center Zhejiang University Hangzhou China

**Keywords:** crohn's disease, observational cohort study, real‐world study, symptomatic strictures, ustekinumab

## Abstract

Despite being a common complication of Crohn's disease (CD), evidence regarding the use of ustekinumab (UST) for treating symptomatic strictures in China remains limited. This study evaluated the effectiveness of UST in this population and identified the predictors of successful therapy. This cohort study enrolled adults with CD initiating UST therapy for symptomatic strictures confirmed by endoscopy or imaging. The primary outcome at Week 52 was continued UST therapy without prohibited adjunctive treatments. A clinical prediction model was developed, and its performance was evaluated for identifying baseline factors associated with treatment success. Of the 73 patients screened between June 2022 and April 2024, 54 were included. At Week 52, 44 (81.5%) achieved treatment success, with most demonstrating improvements in obstructive symptom score, CD Activity Index (CDAI), endoscopic and computed tomography enterography (CTE) findings, and biochemical parameters. Nonsmoking status and C‐reactive protein (CRP) levels predicted treatment success, with the model demonstrating 86.4% discriminative ability. No severe adverse events were reported, except for one case of leukemia (2%) and two cases of serious infections (4%). The study highlighted that UST effectively alleviated intestinal strictures in CD, with most patients achieving symptom remission, improved stricture morphology, and reduced inflammation.

## Graphical abstract

Data on ustekinumab (UST) for symptomatic stricturing Crohn's disease remain scarce in China. This cohort study screened 73 patients from June 2022 to April 2024, of whom 54 were eligible included, with 81.5% achieving 52‐week treatment success. Most attained sustained clinical remission alongside improved inflammation and strictures. Nonsmoking status and low baseline CRP independently predicted favorable outcomes, and UST showed favorable safety.

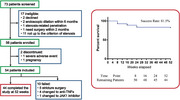

## 1 Introduction

Crohn's disease (CD), a persistent, inflammation‐driven inflammatory bowel disease (IBD), primarily affects the terminal ileum and colon, presenting with heterogeneous clinical manifestations [1]. Approximately 81% of cases present with inflammatory lesions at initial presentation that may evolve into stricturing or penetrating complications, commonly involving the small bowel [2]. Strictures, the most common structural CD‐related complication, arise from inflammation and fibrosis and are detected in ∼20% of cases at early diagnosis [3]. Approximately one‐third of patients develop intestinal strictures within 10 years of diagnosis, and ∼50% require at least one surgical intervention [4].

Traditionally, stricturing CD is managed with endoscopic balloon dilation or surgery, particularly for fibrotic strictures [2]. Pharmacological therapy remains crucial for controlling inflammation in CD and is generally preferred when stenosis is predominantly inflammatory, or when the inflammatory and fibrotic components are indistinguishable, to delay or avoid surgical intervention [5]. The therapeutic effectiveness of anti‐tumor necrosis factor therapies (anti‐TNFs) in stricturing CD remains controversial, as they have been reported to reverse stenosis [6] and increase intestinal obstruction risk [7]. Certain antifibrotic compounds exhibit therapeutic potential in the treatment of CD‐related intestinal strictures [8]. However, no pharmacological agents have been shown to influence stricture development in randomized clinical trials, and patients with established strictures have often been excluded from clinical trials evaluating biological agents.

Ustekinumab (UST), a fully human monoclonal antibody and novel biologic agent for CD, inhibits the p40 component common to both interleukin (IL)‐12 and IL‐23, and received approval in the Netherlands in November 2016 [9]. Its therapeutic benefits in promoting and maintaining remission in moderate‐to‐severe CD are extensively documented [9]. UST inhibits T‐helper 1 and T‐helper 17 signaling cascades, potentially contributing to the alleviation of interstitial fibrosis and ulcer healing. A real‐world study demonstrated its safety and efficacy in CD, with 40% of patients in a highly refractory cohort achieving clinical remission over a 12‐month period [10]. Previous studies have reported the extended therapeutic benefits and safety of UST in CD [11]. Notably, UST has demonstrated effectiveness in the treatment of CD‐related intestinal strictures, resulting in endoscopic remission in two cases of CD with terminal ileal strictures, 24 and 72 weeks post‐therapy [12]. Its therapeutic efficacy was further demonstrated by deep remission achieved in a case of CD‐related refractory stricturing [13]. The latest global consensus suggests that UST can be used for the treatment of CD‐related intestinal fibrostenosis, in both bio‐naïve patients and those who have experienced anti‐TNF failure [14]. However, robust real‐world data pertaining to its therapeutic efficacy in stricturing CD remain limited.

The present single‐center, observational cohort investigation retrospectively evaluated the effectiveness of UST in achieving sustained success in symptomatic stricturing CD and to identify predictors of persistent UST use. Our findings may provide valuable real‐world evidence to inform personalized treatment decisions and optimize the clinical management of this challenging patient population.

## 2 Results

**FIGURE 1:**
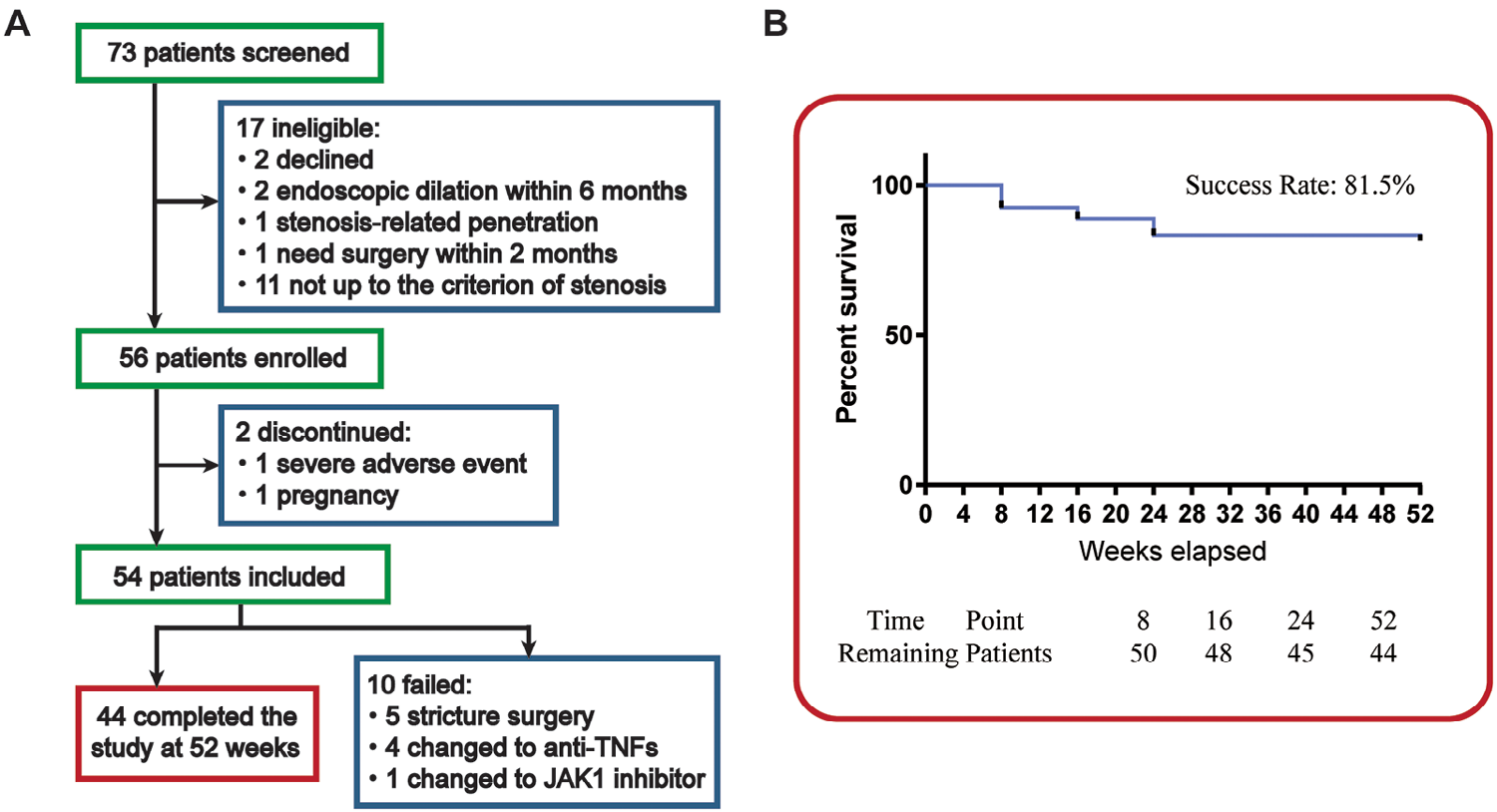
Research process of Crohn's disease (CD) patients with stricturing lesions. (A) Flow chart of study design. anti‐TNFs = anti‐tumor necrosis factor agents. JAK1 inhibitor = Janus Kinase 1 inhibitor. (B) Kaplan–Meier graph showing time to treatment failure in the 54 patients with stricturing CD following the successful response to ustekinumab (UST) over 52 weeks (10 observed failures).

A total of 73 patients with stricturing CD (B2) who received UST therapy were screened in June 2022–April 2024, of whom 56 were enrolled, and 2 subsequently withdrew during the investigation. Seventeen patients were excluded based on the following exclusion criteria: Failure to meet stricture criteria on endoscopy or computed tomography enterography (CTE) (*n* = 11); withdrawal of consent (*n* = 2); history of endoscopic balloon dilatation within the past 6 months (*n* = 2); stenosis‐related perforation before inclusion (*n* = 1); or the need for surgery within 2 months (*n* = 1). The data for the excluded patients were not analyzed, and two patients discontinued the study—one due to pregnancy and one due to a severe adverse event—leaving 54 individuals for the final analysis (Figure 1A).

### 2.1 Baseline Characteristics

Table 1 presents the baseline clinical and demographic features of the study cohort, and Table 2 summarizes the baseline CTE features. Baseline endoscopic examination and intestinal ultrasound revealed that accessible stenosis could not be traversed in 74% of cases (*n* = 40). Baseline tomography data were available for 38 patients (70%), which notably revealed that the majority of dominant strictures were located in the terminal ileum (55%, *n* = 21).

**TABLE 1:** Baseline characteristics of the 54 CD patients with symptomatic small bowel stricture.

Characteristics	*n*, frequency (%) or median (IQR)
Male	39 (72)
Age (years)	24 (21–31)
Time since diagnosis (months)	1 (0–7)
CD phenotype at inclusion (Montreal classification)	
L1 (ileal disease)	25 (46)
L2 (colonic disease)	10 (19)
L3 (ileocolonic disease)	19 (35)
B2 (structuring disease)	54 (100)
*p*	29 (54)
Previous intestinal resection	19 (35)
Previous treatment with infliximab	8 (15)
Smoking history	
Never	48 (88)
Current	3 (6)
Previous	3 (6)
CDAI > 150	24 (46)
CRP (mg/L)	1.8 (0.7–3.7)
FC (µg/g)	1119.3 (368.3–1800)
Dominant stricture location	
Colon	12 (22)
Jejunoileal	7 (13)
Proximal ileum	14 (26)
Terminal ileum (including ileocecal valve)	21 (39)
Obstructive symptoms (CDOS)	
1	5 (9)
2	3 (5)
3	16 (30)
4	3 (5)
5	2 (4)
6	12 (22)
Endoscopy	
Stricture not passable^a^	40 (74)
SES‐CD score of stricture (*n* = 28)^b^	
≤3	17/28 (61)
>3	11/28 (39)
DBE‐CD score (*n* = 33)^c^	
3	6/33 (18)
4	27/33 (82)
IUS	
Limberg classification	
I	6 (11)
II	19 (35)
III	13 (24)
IV	16 (30)
IBUS‐SAS	67.5 (53.3–80.0)
BWT (mm)	5.3 (4.3–6.9)
*i*‐fat	44 (81)
CDS	
Short signals	20 (37)
Long signals inside bowel	15 (28)
Long signals inside and outside bowel	16 (30)
BWS	
Focal (≤ 3cm)	9 (17)
Extensive (> 3cm)	13 (24)

Abbreviations: BWS, bowel wall stratification; BWT, bowel wall thickness; CD, Crohn's Disease; CDAI, Crohn's Disease Activity Index; CDOS, Crohn's disease obstructive score; CDS, Color Doppler imaging signal; CRP, C‐reactive protein; FC, fecal calprotectin; IBUS‐SAS, International Bowel Ultrasound Segmental Activity Score; *i*‐fat, inflammatory mesenteric fat; IUS, intestinal ultrasound.

^a^ Stricture passable endoscopically in 14 patients because of enrollment only identified by tomography features of computed tomography enterography (CTE).

^b^ Data reported for patients with adequate colonoscopy views.

^c^ Data reported for patients with adequate double‐balloon enteroscopy (DBE) views.

**TABLE 2:** Baseline tomography features of the 38 CD patients with symptomatic small bowel stricture(s)^a^ (maximal or more severe characteristic across strictures, except if otherwise indicated).

Characteristics	*n*, frequency (%) or median (IQR)
Location of dominant stricture	
Jejunoileal	4 (11)
Proximal ileum	7 (18)
Terminal ileum	21 (55)
Ileocecum	6 (16)
Max wall thickness (mm)	9.0 (7.3–12)
Dominant stricture length (mm)	45 (27–86)
Luminal diameter in the most narrowed segment (mm)	3.0 (2.0–3.9)
Pre‐stenotic dilation	31 (25–40)
Pre‐stenotic dilation ≥ 30 mm	26 (68)
Dillman ratio	12 (8.02–15)
Morphology	
Single short segment (< 10 cm)	26 (68)
Single long segment (> 10 cm)	6 (16)
Multiple segment	4 (11)
Fistula with dilation	2 (5)
Pattern of enhancement	
Arterial phase	
Homogeneous	10 (26)
Layered	28 (74)
Venous phase	
Homogeneous	13 (34)
Layered	25 (66)
Ulcers	16 (42)
Comb sign	26 (68)
Edema	15 (39)
Inflammatory mass	7 (18)
Abscess	6 (16)
Creeping fat (with fibro‐fatty proliferation)	13 (34)
Fistula	7 (18)
Mesenteric venous thrombosis	3 (8)
Lymphadenectasis (short diameter > 15 mm)	7 (18)

^a^ Stricture not assessable via computed tomography enterography (CTE) in 16 patients: Twelve with colonic strictures (excluded as CTE cannot reliably quantify colonic strictures and the small bowel‐focused evaluation metrics in this table are not applicable to colonic strictures, which are routinely assessed by colonoscopy) and four who did not meet the CTE‐defined stenosis inclusion criteria.

### 2.2 Primary and Secondary Outcomes

By Week 52, 44 of the 54 patients (81.5%) achieved therapeutic success (Figure 1B). Among the remaining patients, five underwent intestinal resection owing to treatment failure, four switched to anti‐TNFs, and one patient transitioned to upadacitinib. UST was discontinued in two patients: One due to a severe adverse event and the other due to pregnancy. The other key secondary outcomes during the follow‐up period (Weeks: 0–52), including the values and longitudinal changes in the CD Obstructive Score (CDOS), CD Activity Index (CDAI), C‐reactive protein (CRP), and fecal calprotectin (FC) levels, as well as endoscopic and imaging findings, are summarized in Table 3.

**TABLE 3:** Primary and secondary outcomes at 52 weeks.

Characteristics	*n*, frequency (%) or median (IQR)
Primary endpoint (*n* = 54)	
Steroid‐free success as UST continuation	44 (81)
Clinical and biochemical secondary endpoints (*n* = 54)	
Treatment failure	10 (19)
Required surgery	5 (9)
Improvement in CDOS ≥ 1 point	28 (52)
CDOS remission^a^	36 (67)
CDOS	1 (0–3)
CDAI < 150	39 (72)
CRP <5 mg/L	37 (69)
Normalization or ≥ 50% reduction in CRP^b^	42 (78)
FC < 100 µg/g	23 (43)
Normalization or ≥ 50% reduction in FC^b^	30 (56)
Normalization or ≥ 50% reduction of CRP and FC	26 (48)
FC, µg/g	153 (47–1160)
CDOS improvement, normal CRP and FC	12 (22)
Endoscopy	
Stricture passable on colonoscopy	11/28 (39)
Stricture passable on double‐balloon enteroscopy	18/33 (55)
Improvement in SES‐CD score of stricture ≥ 1 point^c^	20/28 (71)
Improvement in DBE‐CD score ≥ 1 point^c^	21/33 (64)
CTE (*n* = 38)	
CTE stricture length reduced ≥ 25%	13/38 (34)
Stricture length reduced by ≥ 1 mm	23/38 (61)
Stricture wall thickness improvement ≥ 1 mm	18/38 (47)
Resolution of proximal dilation^d^	20/38 (53)
CTE complete stricture resolution^e^	8/38 (21)
IUS (*n* = 54)	
> 25% improvement in BWT	23 (43)
Normal vascularization (Limberg score ≤ 1)	18 (33)
BWT, mm	4.3(3.3–5.6)

Abbreviations: BWT, bowel wall thickness; CDAI, Crohn's Disease Activity Index; CDOS, Crohn's disease obstructive score; CRP, C‐reactive protein; CTE, computed tomography enterography; FC, fecal calprotectin; IUS, intestinal ultrasound.

^a^ Score of 0–2 considered as symptomatic stricture remission.

^b^ Normalization in CRP (< 5 mg/L) and in fecal calprotectin (< 100 µg/g).

^c^ Data reported for patients with adequate colonoscopy (*n* = 28) and double‐balloon enteroscopy (*n* = 33) views, respectively. Endoscopic endpoints were only assessed in patients with a reachable or assessable stricture.

^d^ Small bowel diameter < 3.0 cm considered as resolution of proximal dilation.

^e^ CTE stricture resolution was defined as normal BWT and luminal diameter with absence of pre‐stenotic dilation, and reduced stricture length by ≥ 1 mm.

**FIGURE 2:**
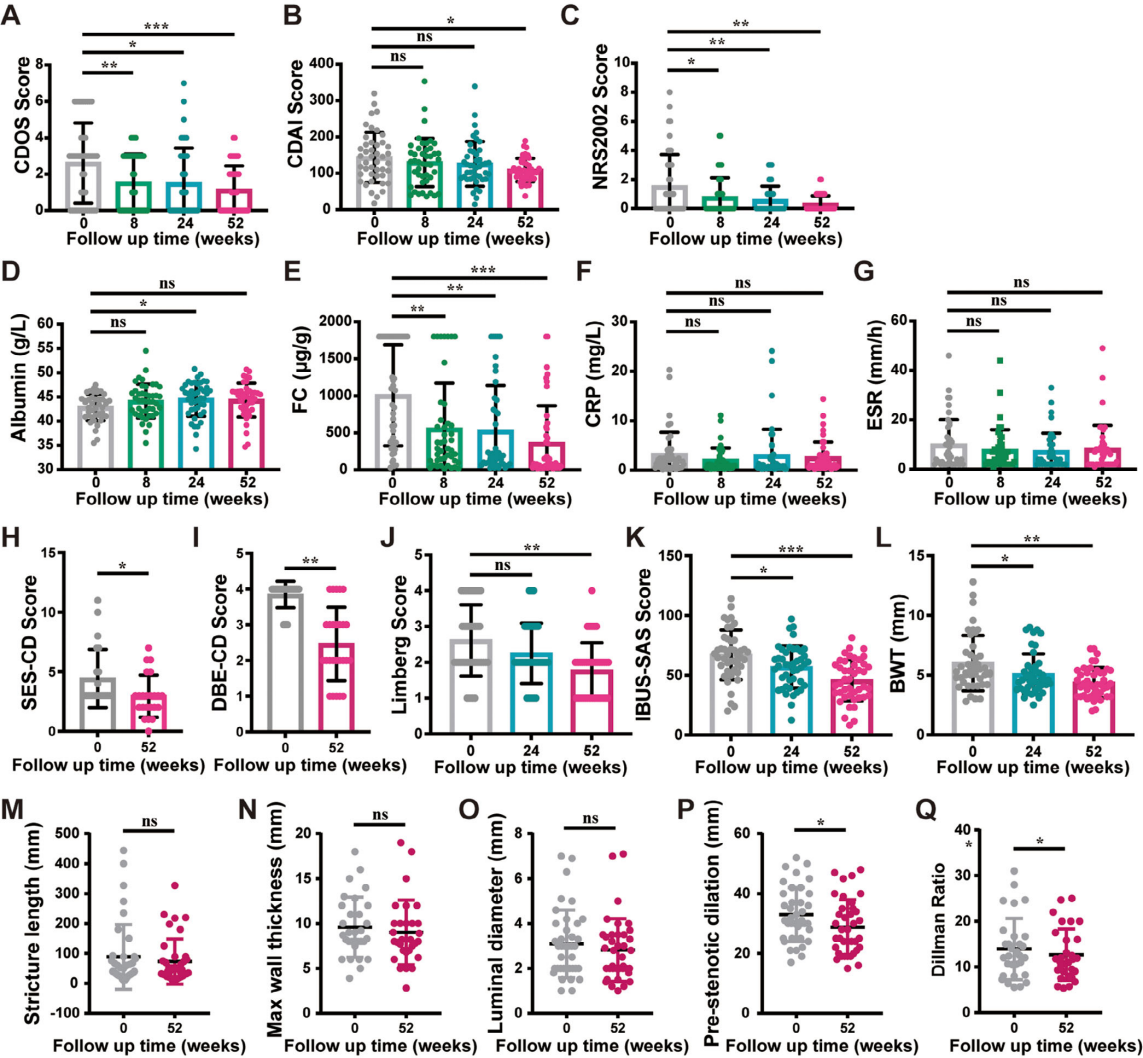
Key outcomes of clinical improvements, inflammatory biochemical markers, and morphology on imaging (endoscopy, CTE, and IUS). (A–C) Clinical outcomes of CDOS, CDAI, and NRS2002 score at 0, 8, 24, 52 weeks (*n* = 44). CDAI = Crohn's Disease Activity Index; CDOS = Crohn's disease obstructive score; NRS2002 = Nutritional Risk Screening 2002. (D–G) Biochemical outcomes of albumin, FC, CRP, and ESR at 0, 8, 24, 52 weeks (*n* = 44). CRP = C‐reactive protein; ESR = erythrocyte sedimentation rate; FC = fecal calprotectin. (H–I) Endoscopy outcomes of SES‐CD (*n* = 23) and DBE‐CD (*n* = 26) score at 0, 52 weeks. DBE‐CD = Double Balloon Endoscopic Score for Crohn's Disease; SES‐CD = Simple Endoscopic Score for Crohn's Disease. (J–L) IUS imaging (*n* = 44) outcomes at 0, 24, and 52 weeks. BWT = bowel wall thickness; IBUS‐SAS = International Bowel Ultrasound Segmental Activity Score; IUS = intestinal ultrasound. (M–Q) CTE imaging outcomes at 0, 52 weeks. CTE = computed tomography enterography.

At the final follow‐up, the CDOS improved by ≥ 1 point in 52% of patients, and 72% achieved a CDAI < 150. The CDOS and CDAI decreased significantly by Week 52 (Figure 2A,B). Nutritional status analysis revealed that, despite modest improvements in serum albumin, the Nutritional Risk Screening 2002 (NRS2002) scores increased significantly at all time points (Figure 2C,D). The CRP levels normalized or decreased by ≥ 50% in 78% of patients, whereas FC levels decreased in 56%. During the follow‐up period, the FC decreased significantly (Figure 2E), with this reduction exhibiting a significant association with treatment response in univariate analysis, but not in the clinical prediction model. The other inflammatory biochemical markers, including CRP and erythrocyte sedimentation rate (ESR), were similarly downregulated (Figure 2F,G).

Imaging, including endoscopy, CTE, and intestinal ultrasonography (IUS), revealed that UST induced substantial improvements in intestinal morphology. Regarding endoscopic stenosis remission, the colonoscope traversed the intestinal narrowing in 11 patients (39%), compared with 28 patients in whom baseline traversal was not possible. In addition, 18 of 33 patients (55%) demonstrated similar improvement during double‐balloon enteroscopy. The Simple Endoscopic Score for CD (SES‐CD) for strictures as well as the Double‐Balloon Endoscopic Score for CD (DBE‐CD) also underwent significant improvements (Figure 2H,I). Among patients with radiological data, 21% exhibited complete resolution of intestinal strictures on CTE (*n* = 8/38). In addition, 61% demonstrated stricture length reduction (*n* = 23/38) and 47% showed improved stricture wall thickness (*n* = 18/38), both associated with increased luminal diameter of the dominant stricture at Week 52 (Figure 2M,O). Proximal dilation resolved in 53% (*n* = 20/38) patients, resulting in a marked reduction in the Dillman ratio (Figure 2P,Q). Limberg classification of IUS data revealed significant reductions in Limberg scores at Week 52 (Figure 2J), and 33% achieved normal vascularization (*n* = 18). The median bowel wall thickness (BWT) decreased significantly to 4.3 mm (IQR: 3.3–5.6) at the final follow‐up, and the International Bowel Ultrasound‐Segmental Activity Score (IBUS‐SAS) also underwent significant reduction at Weeks 24 and 52 (Figure 2K,L).

### 2.3 Subgroup Analysis of Treatment‐Naïve Patients

The baseline demographic and imaging characteristics of treatment‐naïve and returning patients are summarized in Tables S1 and S2. Subgroup analysis revealed no significant differences in the primary and secondary outcomes between treatment‐naïve and previously treated patients (Table S3), indicating that UST exhibited comparable efficacy across patient cohorts irrespective of prior treatment.

### 2.4 Predictors of Treatment Success

Several clinical, laboratory, and imaging factors associated with therapeutic efficacy were identified through univariate analysis (Table 4). Specifically, IBUS‐SAS ≤ 81.8, CRP ≤ 4.4 mg/L, FC ≤ 1234 µg/g, and ESR ≤ 18 mm/h were associated with significantly superior treatment efficacy, with odds ratios (ORs) of 0.222, 0.126, 0.077, and 0.222, respectively, corresponding to 95% confidence intervals (CIs) of 0.052–0.954, 0.028–0.566, 0.009–0.661, and 0.052–0.954, and *p*‐values of 0.043, 0.007, 0.019, and 0.043, respectively. The remaining factors, including age at onset ≤ 31 years, male sex, initial diagnosis, prior surgery, previous use of infliximab (IFX), CDAI ≤ 185, CDOS ≤ 4, Limberg score ≤ 3, BWT ≤ 7.1 mm, and albumin levels ≤ 44.6 g/L, were not significantly associated with differences in treatment efficacy.

**TABLE 4:** Univariate analysis of factors associated with the rate of success.

Factor	Rate of success (%)	Odds ratio of success estimate [95% CI]	*p*
Age > 31 (*n* = 13) vs. ≤ 31 (*n* = 41)	77 vs. 83	0.686 [0.149–3.154]	0.629
Male Female (*n* = 15) vs. Male (*n* = 39)	87 vs. 79	1.677 [0.313–8.995]	0.313
Time since diagnostic (month) >0 (*n* = 31) vs. = 0 (*n* = 23)	81 vs. 83	0.877 [0.217–3.553]	0.854
Previous surgery Yes (*n* = 19) vs. No (*n* = 35)	79 vs. 83	0.776 [0.189–3.179]	0.724
Cigarette Yes (*n* = 6) vs. No (*n* = 48)	50 vs. 85	0.171 [0.029–1.022]	0.053
Previous IFX Yes (*n* = 46) vs. No (*n* = 8)	80 vs. 87	0.587 [0.064–5.398]	0.638
CDAI >185 (*n* = 12) vs. ≤ 185 (*n* = 42)	75 vs. 83	0.600 [0.129–2.794]	0.515
CDOS >4 (*n* = 12) vs. ≤ 4 (*n* = 42)	75 vs. 83	0.600 [0.129–2.794]	0.515
Limberg score >3 (*n* = 16) vs. ≤ 3 (*n* = 38)	69 vs. 87	0.333 [0.081–1.372]	0.128
IBUS‐SAS >81.8 (*n* = 13) vs. ≤ 81.8 (*n* = 41)	62 vs. 88	0.222 [0.052–0.954]	0.043
BWT >7.1 (*n* = 13) vs. ≤ 7.1 (*n* = 41)	77 vs. 83	0.686 [0.149–3.154]	0.629
CRP >4.4 (*n* = 13) vs. ≤ 4.4 (*n* = 41)	54 vs. 90	0.126 [0.028–0.566]	0.007
Albumin >44.6 (*n* = 13) vs. ≤ 44.6 (*n* = 41)	77 vs. 83	0.686 [0.149–3.154]	0.629
FC >1234 (*n* = 27) vs. ≤ 1234 (*n* = 27)	67 vs. 96	0.077 [0.009–0.661]	0.019
ESR >18 (*n* = 13) vs. ≤ 18 (*n* = 41)	62 vs. 88	0.222 [0.052–0.954]	0.043

Abbreviations: BWT, bowel wall thickness; CDAI, Crohn's Disease Activity Index; CDOS, Crohn's disease obstructive score; CRP, C‐reactive protein; ESR, erythrocyte sedimentation rate; FC, fecal calprotectin; IBUS‐SAS, International Bowel Ultrasound Segmental Activity Score; IFX, infliximab.

### 2.5 Clinical Prediction Model

**FIGURE 3:**
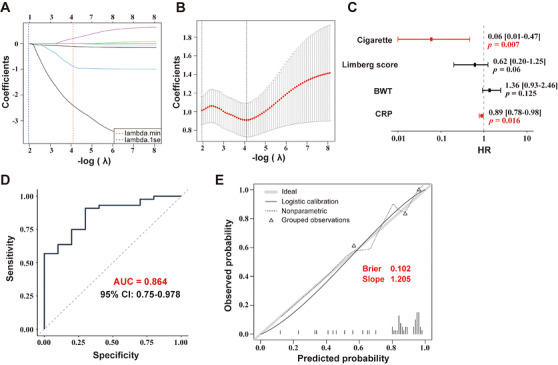
Clinical prediction model and performance evaluation of factors associated with treatment success from multivariate analysis. (A, B) Coefficient path and cross validation of the LASSO regression. (C) Forest plot of the Firth's penalized logistic regression. (D) Receiver operating characteristic curve of the prediction model. (E) Calibration plot of the prediction model.

The most relevant predictors of treatment success were identified through Least Absolute Shrinkage and Selection Operator (LASSO)‐penalized logistic regression. Among the eight candidate variables, four, including smoking status, Limberg score, BWT, and CRP, retained nonzero coefficients at the optimal penalty parameter (*λ* = lambda.min; Figure 3A,B), and were further analyzed using Firth's penalized logistic regression. Smoking (OR = 0.06; 95% CI: 0.01–0.47; *p* = 0.007) as well as CRP level (OR = 0.89; 95% CI: 0.78–0.98; *p* = 0.016) emerged as independent negative predictors of therapeutic success, whereas the Limberg score and BWT were insignificant. The results are presented in the forest plot (Figure 3C).

### 2.6 Model Performance Evaluation

The four variables selected via LASSO regression were incorporated into the Firth logistic regression model. The predictive model displayed strong discriminatory potential (apparent AUC: 0.864; 95% CI: 0.750–0.978; Figure 3D) and satisfactory calibration (Brier score: 0.102; calibration slope: 1.205; Figure 3E). Internal validation using 500 bootstrap resamples yielded an optimism‐corrected AUC of 0.824, confirming the robust internal validity of the model.

### 2.7 Safety Assessments

Adverse events were observed in 15 (27%) of the 56 patients (Table 5). Overall, UST was discontinued in six patients (11%) due to these adverse events. Among them, one patient, who was diagnosed with leukemia during a COVID‐19 infection, exhibited persistently low white blood cell counts 8 weeks after initiating UST. This patient underwent a bone marrow transplantation, and UST treatment was discontinued 3 months later. Another patient became pregnant during the study, and treatment was subsequently discontinued. In addition, 9% required surgical intervention for CD, and 13% were hospitalized due to CD‐related complications. Among these patients, 10 (18%) developed infections, including one case with severe abdominal pain and another case of severe acute diarrhea. In addition, two patients experienced fever and nausea, three presented with arthralgia, four reported dizziness and headache, and five experienced fatigue. No mortality was recorded during the study.

**TABLE 5:** Adverse events in 56 patients registered during the study.

Characteristics	*n*, frequency (%)
Patients with at least one adverse event	15 (27)
Adverse events leading to discontinuation of UST	6 (11)
Patients with adverse events	
Surgery for CD	5 (9)
Endoscopic dilation	0 (0)
Hospitalization for CD complications	7 (13)
Infection^a^	10 (18)
Severe infection^b^	2 (4)
Severe abdominal pain	3 (5)
Fever and nausea	2 (4)
Severe acute diarrhea	1 (2)
Arthralgia	3 (5)
Dizziness and headache	4 (7)
Fatigue	5 (9)
Leukemia^c^	1 (2)
Death	0 (0)

^a^ Infection and severe infection rate excluded developing COVID‐19 while the wide circulation of Omicron variants ensued in China after the end of “zero COVID” strategy since December 2022.

^b^ Severe infection was defined as an infection requiring hospitalization or treatment with intravenous antibiotics or antiviral agents.

^c^ The patient diagnosed with leukemia 8 weeks after UST initiation with consistently low white blood cells during COVID‐19 infection, UST discontinued while bone marrow transplantation performed 3 months later.

## 3 Discussion

This investigation aimed to advance the understanding of therapeutic responses in CD‐associated enterostenosis, evaluate the real‐world clinical benefits and safety of UST, and identify clinical and imaging predictors of treatment success with UST. Our findings demonstrate that UST achieved sustained treatment success in 81.5% of patients with symptomatic strictures at Week 52, accompanied by a relatively low incidence of serious adverse events.

Although CD‐related strictures are typically predominantly fibrotic and irreversible, they are frequently accompanied by inflammation, which may independently contribute to fibrosis [15]. Previous studies correlating imaging findings with histological observations have reported inconsistent results regarding the relationship between inflammation and fibrosis [16]. A retrospective study comparing 53 magnetic resonance enterographies (MREs) with pathological analyses of surgical specimens reported an association between chronic inflammation accumulation and increased fibrosis [17]. Our study characterized the structural reversibility of intestinal strictures posttreatment with UST, providing evidence for the presence of reversible components. In the Chinese cohort included in this study, most strictures were located in the small bowel, resulting in low baseline endoscopic scores. Although the distal segments of non‐traversable strictures could not be assessed based on SES‐CD, the complete resolution of colonic strictures on colonoscopy further verified stricture reversal. However, high costs, poor patient tolerance to invasive enteroscopy and reluctance to receive repeated radiological CTE scans led to incomplete imaging data, thus our core conclusions were based primarily on composite clinical endpoints rather than mucosal or structural healing outcomes.

The primary objective of pharmacological therapy in CD with intestinal strictures is to determine whether it can prevent or delay surgical intervention. Previous retrospective studies have demonstrated that anti‐TNF therapy can effectively minimize stricture‐related surgeries [18, 19]. For instance, a large retrospective study involving 136 patients with strictures and a prospective study of 97 patients reported that up to 50% of patients required surgery within 4 years (medium term), despite receiving optimal pharmacological therapy [6]. In the present study, the 12‐month surgical intervention rate was 9.3%, which is substantially lower than those reported in historical control cohorts. However, the precise contribution of UST to this outcome remains uncertain owing to our observational and uncontrolled study design. Although the ability of UST to modify the disease trajectory of CD cannot be definitively established, our findings indicate this potential, especially considering the latest global consensus indicating that UST may be appropriate for reversing intestinal strictures [14].

UST inhibits IL‐12/IL‐23 signaling, which is a key contributor to chronic intestinal inflammation. By suppressing chronic inflammation, UST further mitigates inflammation‐driven intestinal fibrosis, the primary mechanism underlying intestinal stricture formation in CD. The therapeutic effects of UST on intestinal strictures are likely mediated by a delicate balance between the amelioration of interstitial fibrosis and enhanced ulcer healing [13, 20]. The therapeutic effects of UST on intestinal stricture resolution are attributed to the inhibition of chronic intestinal inflammation, leading to the restoration of local immune homeostasis [13]; suppression of Th1 cell activity, thereby reducing TGF‐*β*‐mediated profibrotic signaling; and modulation of Th17 cell function, which consequently downregulates IL‐17/IL‐22 expression [21]. Collectively, these mechanisms inhibit myofibroblast formation, epithelial/endothelial‐mesenchymal transition (EMT/EndoMT), and extracellular matrix (ECM) deposition, thereby attenuating intestinal stricture progression [22, 23].

Although several studies have reported the real‐world efficacy of UST [9, 24], this investigation represents the first retrospective assessment of its therapeutic efficacy in symptomatic stricturing CD within a Chinese patient cohort. Our findings revealed significant improvements in all key inflammatory and structural parameters at Week 52. Subgroup analyses revealed comparable primary outcomes between treatment‐naïve and previously treated patients, providing preliminary evidence supporting the utility of UST in treatment‐naïve individuals—an understudied population in clinical practice. Notably, our cohort reflects unique real‐world contexts, including patients initiated on UST promptly after Medicare approval, which likely enriched the cohort with refractory cases. In addition, the preferential use of intravenous (IV) UST, owing to its cost‐effectiveness, potentially contributed to the high success rate observed herein. UST demonstrated a favorable safety profile, with the rates of serious adverse events (2%) and severe infections (4%) consistent with previous real‐world data [9, 24, 25].

The relatively low baseline CRP and ESR levels reflect two key characteristics of our cohort. First, in accordance with Chinese clinical practice, patients received 2 weeks of conventional therapy, including exclusive enteral nutrition (EEN) and low‐dose corticosteroids, prior to biologic therapy, which reduced systemic inflammation at enrollment. Second, most patients had small bowel stricturing, a phenotype frequently with mild inflammatory markers despite moderate‐to‐severe disease activity confirmed by clinical and radiologic assessments. In addition, low baseline CRP levels indicate a fibrosis‐predominant rather than an inflammation‐predominant phenotype, which typically responds more slowly to anti‐inflammatory biologics, including UST, thereby explaining the negative correlation between baseline CRP levels and therapeutic response. Our treatment success was defined as sustained UST use rather than full mucosal or structural healing, precluding definite confirmation of its direct anti‐fibrotic activity as most current indicators [23]. Clinically, distinct from anti‐TNFs that may exacerbate stricture obstruction [7], UST exerts milder regulatory effects, hinting at its potential anti‐fibrotic action independent of canonical inflammatory pathways for further basic and clinical validation.

We evaluated the predictive factors associated with the efficacy of UST for managing CD‐related intestinal strictures. Smoking, a well‐established environmental risk factor for CD, has been associated with increased stricture‐related complications [26]. A systematic review and meta‐analysis reported that smokers receiving anti‐TNF therapy were less likely to achieve a clinical response than nonsmokers [27]. In addition, the history of smoking is a key risk factor for recurring intestinal strictures following endoscopic dilation or urethroplasty [26, 28]. Consistent with previous reports, the present study revealed that patients without a history of smoking exhibited a superior response to UST treatment than smokers. Similarly, individuals exhibiting lower CRP levels demonstrated a superior response to UST, which aligns with existing literature [29]. Although Hyams et al. reported that pediatric patients with CD and elevated CRP levels are more responsive to adalimumab therapy than those with low CRP levels, few studies have specifically investigated symptomatic stricture remission. These findings are critical for clarifying the contributions of UST to the management algorithm for CD‐related intestinal structuring.

Our primary endpoint at Week 52 was consistent with one of the two primary endpoints used in the ICC registry studies [24, 30]. An earlier endpoint would be insufficient for evaluating the responses to UST therapy in stricturing CD. We therefore prioritized treatment‐failure–free survival as a clinically robust endpoint, in line with the latest global consensus on stricturing CD [14], which recommends this composite outcome as a core efficacy endpoint and defines freedom from endoscopic or surgical intervention as a clinical hard endpoint for biologic therapy. Endoscopic improvement was designated a secondary endpoint, partly because most patients decline invasive and costly small‐bowel enteroscopy. Supported by strong patient compliance and shared clinician–patient decision‐making, the endpoints selected in our study minimized major confounders. These composite endpoints have been widely implemented in real‐world studies on biologic therapies for CD [6, 14, 19, 31], thus supporting the validity of our study design.

The robustness of our study lies in its stringent evaluation criteria and systematic follow‐up, with all patients managed consistently by a dedicated IBD team. The substantial size of our cohort, encompassing nationwide coverage, enabled the development of a representative sample reflective of routine clinical care. The characteristics of the patient cohort enabled the documentation of clinically meaningful benefits and the adverse event profile of UST. To minimize bias, patients with insufficient follow‐up time were censored, irrespective of their clinical response. Furthermore, we are actively recruiting patients for prospective studies, which may inform future randomized trials designed to investigate the true clinical benefits and safety of various therapeutic strategies with largely comparable baseline characteristics.

The present research is subject to numerous limitations. First, this research represents a retrospective observational study without a control group. Nevertheless, European guidelines recommend surgical intervention for all such patients, irrespective of specific criteria [32]. Accordingly, controlled trials comparing UST with surgical management are warranted to determine the optimal strategy for preserving the quality of life in CD. Second, the subgroup analysis of treatment‐naïve patients was limited by the small cohort size and the correspondingly low statistical power. Third, the predictive factors identified herein require further validation in independent patient cohorts. Future multicenter prospective studies are needed to validate our findings in larger populations, compare UST with other biologics in stricturing CD, and further investigate treatment‐naïve patients to optimize clinical management.

Finally, as a single‐arm retrospective cohort study, prior short‐term EEN and low‐dose corticosteroid use is an inherent confounding factor for assessing UST efficacy. Notably, all prior conventional interventions were administered as short‐term transitional therapies, with 2‐week EEN and full steroid discontinuation within 2 months after UST initiation. Given UST's delayed efficacy onset, they exerted limited impacts on our 52‐week primary endpoints. This pretreatment pattern complies with clinical practice and domestic insurance policies. Although we acknowledge its potential confounding effects, long‐term follow‐up design ensures sustained benefits are predominantly attributed to UST intervention.

In conclusion, the favorable benefits and tolerability of UST are demonstrated in a real‐world cohort with symptomatic stricturing CD. The findings revealed that approximately 80% of patients achieved sustained clinical success at Week 52, accompanied by the significant downregulation of inflammatory markers. These findings support UST as a potential therapeutic option for this challenging CD‐related complication. Ongoing prospective studies aim to further elucidate its precise role in managing CD‐related intestinal strictures.

## 4 Materials and Methods

### 4.1 Research Methodology

This observational cohort study was performed retrospectively at The Second Affiliated Hospital of Zhejiang University School of Medicine in June 2022–April 2024. All cases were derived from the precise evaluation, Personalized Risk Stratification, and Targeted Treatment (PRIST) Cohort, established in July 2019 at our IBD center. The research received ethical approval from the Institutional Ethics Committee (reference: 2023‐0004) and was carried out following the STROBE reporting guidelines (Appendix S1).

### 4.2 Patient Cohort

Patients aged 18–65 years with moderate‐to‐severe CD, one or more luminal strictures, and obstructive symptoms, who were treated with UST, were enrolled [6, 14, 19, 33]. Intestinal strictures were defined as > 50% luminal narrowing, > 25% increased BWT, and > 2.5 cm pre‐stricture dilation on CTE [34], or as non‐traversable strictures identified on endoscopy. Individuals meeting any of these exclusion criteria were ineligible for inclusion [6, 14, 18, 33]: Severe disease requiring urgent surgery within 2 months, including acute severe intestinal obstruction, perforation, intra‐abdominal abscess, or adhesions; inability to resume oral intake despite EEN for ≥ 2 months; prior exposure to IL‐23 antagonists within the preceding 12 months; relative contraindications to biologic therapy (active tuberculosis, myocardial infarction, heart failure, or demyelinating neurological disorders) within the past 5 years; and contraindications to CTE, such as allergy to contrast medium. In addition, individuals undergoing chemotherapy or radiation therapy for malignant disease; had short bowel syndrome or severe malabsorption following multiple abdominal surgeries [33]; with active gastrointestinal bleeding, severe hepatic or renal dysfunction, active bacterial or viral infection, or shock; were pregnant or lactating; or had unstable vital signs or end‐stage disease were excluded (Appendix S2).

All patients received weight‐based IV UST infusion at baseline (260, 390, and 520 mg for ≤ 55, 55–85, and ≥ 85 kg, respectively), followed by intensive 260 mg IV UST infusion at Week 8. Patients with normalized inflammatory markers (CRP and FC) by Week 8 were transitioned to maintenance therapy with 90 mg subcutaneous (SC) or 130 mg IV UST every 8 weeks. Cases with abnormally elevated inflammatory marker levels continued to receive 260 mg IV UST infusion every 8 weeks until normalization on subsequent re‐evaluation. Patients with prior exposure to biologic therapies other than IL‐23 antagonists were also included.

### 4.3 Study Procedures

Demographic data, disease‐related parameters (location, extent, behavior, and other relevant characteristics), stricture status, imaging findings, inflammatory marker levels, treatment history, and complication profiles were systematically collected from all patients. Any missing data were verified through direct patient interviews.

Imaging assessments included endoscopy, CTE, and IUS, performed at baseline, and independently reviewed by two IBD specialists, one radiologist, and three sonologists. A dedicated session was held with all specialists to systematically evaluate the stricture criteria identified in the CD cohort. Inflammation at the stricture site was confirmed through endoscopy or CTE, and in patients with multiple strictures, the dominant stricture was identified and reassessed at Week 52.

Clinical, inflammatory, and nutritional assessments, including the measurement of CDAI, FC and CRP levels, ESR, NRS2002, and serum albumin levels, were performed at baseline and at Weeks 8, 24, and 52. Nutritional risk was assessed using NRS2002, a standardized tool developed by the European Society for Parenteral and Enteral Nutrition (ESPEN), based on impaired nutritional status, disease severity, and age, and a total score ≥ 3 is indicative of nutritional risk [35]. CDOS [6] was employed to assess and grade the severity of intestinal obstruction symptoms on a scale of 0–6, based on reports of obstructive pain, related clinical signs, dietary limitations, and hospitalizations, and recorded at Weeks 0, 12, 24, and 52. CDOS was systematically assessed and documented for all patients with stricturing CD at each scheduled outpatient follow‐up visit. A comprehensive clinical database was subsequently established at our center for this patient phenotype using the aforementioned standardized evaluation protocol. Scoring was performed by experienced IBD specialists to ensure consistency and reliability.

### 4.4 Outcomes

The primary outcome, evaluated at Week 52, was defined as successful continued treatment with UST [6, 14, 19, 31], in the absence of the any of the following: (a) Rescue therapies, including corticosteroid therapy beyond 8 weeks post‐enrollment, parenteral nutrition, or additional biological agents; (b) endoscopic dilations; (c) intestinal surgeries; (d) serious adverse effects necessitating UST discontinuation; or (e) withdrawal from the investigation for any cause. The secondary outcomes included an improvement in the obstructive symptoms, indicated by a ≥ 1‐point increase in CDOS, changes in imaging and endoscopic findings, requirements for surgery or UST discontinuation, and adverse events.

An improvement in endoscopic findings was defined by increased luminal diameter, successful passage of a standard colonoscope, and lower SES‐CD or DBE‐CD. Improved CTE findings were defined by the resolution of prestenotic dilatation, stricture resolution (normal BWT and luminal diameter without prestenotic dilatation), and a ≥ 1 mm reduction in stricture length. Improved IUS was defined as the normalization (< 3.0 cm) or a ≥ 50% reduction in prestenotic dilatation, a > 25% decrease in BWT, and lower IBUS‐SAS scores. Clinical improvement was assessed based on CDAI remission (CDAI < 150), NRS2002, as well as by altered CRP and FC levels at Weeks 8, 24, and 52. The baseline factors were analyzed to identify predictors of treatment success at Week 52.

The safety outcomes included adverse events, infections, and CD‐related hospitalizations and surgeries. Severe infections were defined as those requiring hospitalization or IV antimicrobial therapy. Adverse events resulting in UST discontinuation were also documented.

### 4.5 Treatment‐Naïve Subgroup

Treatment‐naïve patients were defined as individuals who initially presented to our Department of Gastroenterology, had not previously received standardized treatment for CD, including glucocorticoids, immunosuppressants, or biologic agents, and who received UST for the first time in our study for symptomatic stricturing CD. A comparative analysis was performed between this subgroup and patients who had previously received standardized treatment for CD and were subsequently initiated on UST therapy due to disease progression or treatment failure.

### 4.6 Statistical Analysis

SPSS (v21.0; IBM Corp., Armonk, NY, USA) and R (v4.5.2; R Foundation for Statistical Computing, Vienna, Austria) were implemented for the statistical analyses. The continuous variables have been reported as the mean ± standard deviation (SD) or median with interquartile range (IQR). The group differences were determined based on unpaired *t*‐tests and Mann–Whitney *U* tests. The categorical variables have been reported as percentages, and *χ* ^2^ and Fisher's exact tests were implemented for estimating between‐group differences. Any missing binary variables were coded as “no improvement,” whereas missing continuous data were imputed using the last available measurement, or baseline values when subsequent measurements were unavailable. A two‐sided *p* < 0.05 denoted statistical significance.

### 4.7 Univariate Analysis

The baseline characteristics and potential predictive factors were compared between the treatment success and failure groups. Continuous variables were categorized by quartiles and reported as the mean ± SD or median, whereas categorical variables have been reported as counts or percentages. Univariate comparisons were performed using appropriate statistical analyses, including Student's *t*‐tests, Mann–Whitney *U* tests, or *χ* ^2^ tests.

### 4.8 Predictor Variables and Model Development

The predictor variables were selected using LASSO logistic regression with the “Glmnet” package in R. The optimal regularization parameter (*λ*) was estimated through 10‐fold cross‐validation using the smallest value of *λ* (lambda.min). Variables with nonzero coefficients at the optimal *λ* were retained as candidate predictors. Considering the limited sample size and separation risk, Firth's penalized logistic regression, implemented in the “logistf” package in R, was applied for final model construction. ORs with 95% CIs were calculated for each predictor.

### 4.9 Model Evaluation and Validation

The discriminative ability of the model was evaluated based on the area under the ROC curve (AUC). Model calibration was assessed with a calibration plot and Brier score. To estimate its internal validity and correct for potential optimism arising from overfitting, bootstrap resampling with 500 iterations was performed. An optimism‐corrected AUC was calculated and reported.

## Author Contributions

J.L. drafted the manuscript. S.L., S.H., W.H., D.J., and Y.C. revised the manuscript. Y.C. supervised the project. All authors approved the final version.

## Funding

Yan Chen has received speaker honoraria from Takeda China, Xian Janssen, and AbbVie China, as well as research funding from Johnson & Johnson Innovative Medicine. Jingwen Liu received research funding from the Provincial Natural Science Foundation of Zhejiang Province [LQN26H160014].

## Ethics Statement

Ethical approval for this research was provided by the Institutional Review Board of The Second Affiliated Hospital, Zhejiang University School of Medicine (Reference: 2023‐0004) for clinical experiments.

## Conflicts of Interest

The authors declare no conflicts of interest.

## Supporting information




**Table S1**: Baseline characteristics of treatment‐naïve and previously treated subgroups in patients with symptomatic stricturing CD.
**Table S2**: Baseline tomography features of the treatment‐naïve and previously treated subgroups in patients with symptomatic stricturing CD^*^ (maximal or more severe characteristic across strictures, except if otherwise indicated).
**Table S3**: Primary and secondary outcomes of subgroups at 52 weeks.
**Supplemental Appendix 1**: STROBE Statement—Checklist of items that should be included in reports of *cohort studies*.
**Supplemental Appendix 2**: Inclusion and Exclusion Criteria.

## Data Availability

All data are incorporated into the article and its online Supporting Information.
