# Supplementary material for: Sustained Treatment Success With Ustekinumab in Symptomatic Stricturing Crohn's Disease: A Retrospective Single‐Arm Observational Cohort Study
Source: MedComm (2020). 2026 Jun 17;7(7):e70824. doi: 10.1002/mco2.70824 (PMC13273843; doi:10.1002/mco2.70824)
Supplement: Supplementary file 1 — Table S1: Baseline characteristics of treatment‐naïve and previously treated subgroups in patients with symptomatic stricturing CD. Table S2: Baseline tomography features of the treatment‐naïve and previously treated subgroups in patients with symptomatic stricturing CD* (maximal or more severe characteristic across strictures, except if otherwise indicated). Table S3: Primary and secondary outcomes of subgroups at 52 weeks. Supplemental Appendix 1: STROBE Statement—Checklist of items that should be included in reports of cohort studies. Supplemental Appendix 2: Inclusion and Exclusion Criteria. [file MCO2-7-e70824-s001.docx]

**Sustained Treatment Success with Ustekinumab in Symptomatic Stricturing Crohn’s Disease: A Retrospective Single-Arm Observational Cohort Study**

Jingwen Liu ^1,#^, Wen Hu^2,#^, Shuyan Li^3^, Shurong Hu^1^, Deming Jiang^4,*^, Yan Chen^1,5,*^

1. Department of Gastroenterology, The Second Affiliated Hospital, Zhejiang University School of Medicine, Hangzhou, China
2. Department of Gastroenterology, The First Affiliated Hospital of Zhejiang Chinese Medical University (Zhejiang Provincial Hospital of Chinese Medicine), Hangzhou, China
3. Department of Nursing, The Second Affiliated Hospital, Zhejiang University School of Medicine, Hangzhou, China
4. Laboratory Animal Center, Zhejiang University, Hangzhou, China
5. Lead contact

**^#^** These two authors contribute equally to this study.

**^*^ Corresponding Author:**

Yan Chen, Department of Gastroenterology, the Second Affiliated Hospital, School of Medicine, Zhejiang University, Hangzhou, China. E-mail: [chenyan72_72@zju.edu.cn](mailto:chenyan72_72@zju.edu.cn)

Deming Jiang, Laboratory Animal Center, Zhejiang University, Hangzhou, China, Email: gemnjiang@zju.edu.cn

**Table S1 Baseline characteristics of treatment-naïve and previously treated subgroups in patients with symptomatic stricturing CD**

| Characteristics (n, frequency [%] or median [IQR]) | Treatment-naïve subgroup (n=25) | previously treated subgroup (n=29) |
| --- | --- | --- |
| Male | 19 (76) | 20 (69) |
| Age (years) | 23 (20-28) | 28 (22-41) |
| Time since diagnosis (months) | 0 (0-1) | 22 (6-47) |
| CD phenotype at inclusion (Montreal classification) | | |
| L1 (ileal disease) | 12 (48) | 13 (45) |
| L2 (colonic disease) | 3 (12) | 7 (24) |
| L3 (ileocolonic disease) | 10 (40) | 9 (31) |
| B2 (structuring disease) | 25 (100) | 29 (100) |
| p | 12 (48) | 17 (59) |
| Previous intestinal resection | 0 (0) | 19 (66) |
| Previous treatment with infliximab | 0 (0) | 8 (28) |
| Smoking history | | |
| Never | 23 (92) | 25 (86) |
| Current | 2 (8) | 1 (3) |
| Previous | 0 (0) | 3 (10) |
| CDAI > 150 | 13 (52) | 11 (38) |
| CRP (mg/L) | 2.1 (1.2–6.9) | 3.3 (2.0–30.6) |
| FC (μg/g) | 1585.32  (529.3-1800) | 1792.9  (688.81-1800) |
| Dominant stricture location | | |
| Colon | 5 (20) | 7 (24) |
| Jejunoileum | 4 (16) | 3 (10) |
| Proximal ileum | 6 (24) | 8 (28) |
| Terminal ileum (including ileocecal valve) | 10 (40) | 11 (38) |
| Obstructive symptoms (CDOS) | | |
| 1 | 4 (16) | 1 (3) |
| 2 | 2 (8) | 1 (3) |
| 3 | 5 (20) | 11 (38) |
| 4 | 1 (4) | 2 (7) |
| 5 | 1 (4) | 1 (3) |
| 6 | 4 (16) | 8 (28) |
| Endoscopy | | |
| Stricture not passable* | 17 (68) | 23 (79) |
| SES-CD score of stricture^†^ | | |
| ≤ 3 | 9/13 (69) | 8/15 (53) |
| > 3 | 4/13 (31) | 7/15 (47) |
| DBE-CD score^‡^ | | |
| 3 | 4/18 (22) | 2/15 (13) |
| 4 | 14/18 (78) | 13/15 (87) |
| IUS | | |
| Limberg classification | | |
| I | 4 (16) | 2 (7) |
| II | 12 (48) | 7 (24) |
| III | 4 (16) | 9 (31) |
| IV | 5 (20) | 11 (38) |
| IBUS-SAS | 64.8 (63.6-87.4) | 69.4 (52.2-90.2) |
| BWT (mm) | 5.4 (4.1-7.8) | 5.5 (4.2-8.8) |
| i-fat | 23 (92) | 21 (72) |
| CDS | | |
| Short signals | 13 (52) | 7 (24) |
| Long signals inside bowel | 3 (12) | 12 (41) |
| Long signals inside & outside bowel | 6 (24) | 10 (34) |
| BWS | | |
| Focal (≤ 3 cm) | 4 (16) | 5 (17) |
| Extensive(> 3 cm) | 5 (20) | 8 (28) |

CDAI=Crohn’s Disease Activity Index. CRP=C-reactive protein. FC=fecal calprotectin. CDOS=Crohn’s disease obstructive score. IUS=intestinal ultrasound. IBUS-SAS=International Bowel Ultrasound Segmental Activity Score. BWT=bowel wall thickness. i-fat=inflammatory mesenteric fat. CDS=Colour doppler imaging signal. BWS=bowel wall stratification.

* Stricture passable endoscopically in 8 patients from the treatment-naïve subgroup and 6 from the previously treated subgroup because of enrollment only identified by tomography features of computed tomography enterography (CTE).

† Data reported for patients with adequate colonoscopy views.

‡ Data reported for patients with adequate double-balloon enteroscopy (DBE) views.

**Table S2 Baseline tomography features of the treatment-naïve and previously treated subgroups in patients with symptomatic stricturing CD* (maximal or more severe characteristic across strictures, except if otherwise indicated)**

| Characteristics (n, frequency [%] or median [IQR]) | Treatment-naïve subgroup (n=20) | previously treated subgroup (n=18) |
| --- | --- | --- |
| Location of dominant stricture |  |  |
| Jejunoileum | 3 (15) | 1 (5) |
| Proximal ileum | 4 (20) | 3 (17) |
| Terminal ileum | 11 (55) | 10 (56) |
| Ileocecum | 2 (10) | 4 (22) |
| Max wall thickness (mm) | 8.65 (6.2-10.7) | 9.4 (7.5-13.8) |
| Dominant stricture length (mm) | 33.5 (18.2-80.5) | 67.5 (31.2-130) |
| Luminal diameter in the most narrowed segment (mm) | 2.5 (1.5-3.6) | 3.3 (2.3-4.6) |
| Pre-stenotic dilation | 30.5 (22-37) | 31.5 (27-43) |
| Pre-stenotic dilation ≥ 30 mm | 12 (60) | 14 (78) |
| Dillman ratio | 13.5 (8.25-18.8) | 10 (7.8-14.1) |
| Morphology |  |  |
| Single short segment (<10 cm) | 16 (80) | 10 (55) |
| Single long segment (>10 cm) | 3 (15) | 3 (17) |
| Multiple segment | 1 (5) | 3 (17) |
| Fistula with dilation | 0 (0) | 2 (11) |
| Pattern of enhancement |  |  |
| Arterial phase |  |  |
| Homogeneous | 8 (40) | 2 (11) |
| Layered | 12 (60) | 16 (89) |
| Venous phase |  |  |
| Homogeneous | 7 (35) | 6 (33) |
| Layered | 13 (65) | 12 (67) |
| Ulcers | 9 (45) | 7 (39) |
| Comb sign | 11 (55) | 15 (83) |
| Edema | 6 (30) | 9 (50) |
| Inflammatory mass | 3 (15) | 4 (22) |
| Abscess | 2 (10) | 4 (22) |
| Creeping fat (with fibro-fatty proliferation) | 5 (25) | 8 (44) |
| Fistula | 2 (10) | 5 (28) |
| Mesenteric venous thrombosis | 1 (5) | 2 (11) |
| Lymphadenectasis (short diameter > 15 mm) | 3 (15) | 4 (22) |

* Stricture not assessable via computed tomography enterography (CTE) in 5 patients from the treatment-naïve subgroup and 11 from the previously treated subgroup: 3 treatment‑naïve and 9 previously treated patients observed colonic strictures, while 2 treatment‑naïve and 2 previously treated patients did not meet the stricture inclusion criteria defined by CTE.

**Table S3 Primary and secondary outcomes of subgroups at 52 weeks.**

| Characteristics (n, frequency [%] or median [IQR]) | Treatment-naïve subgroup (n=25) | previously treated subgroup (n=29) | OR, median or mean difference  (95% CI) | p value |
| --- | --- | --- | --- | --- |
| Primary endpoint | | | | |
| Steroid-free success as UST continuation | 21 (84) | 23 (79) | 1.37 (0.339-25.535) | 0.659 |
| Clinical and biochemical secondary endpoints | | | | |
| Treatment failure | 4 (16) | 6 (21) | 0.730  (0.181-.951) | 0.659 |
| Required surgery | 3 (12) | 2 (7) | 1.841 (0.282-12.012) | 0.524 |
| Improvement in CDOS ≥1 point | 12 (48) | 16(55) | 0.750 (0.257-2.193) | 0.599 |
| CDOS remission^†^ | 17 (68) | 19(66) | 0.570 (0.190-1.707) | 0.315 |
| CDOS | 1 (0-2) | 0(0-2) | 0.0298 (-0.7711-0.8307) | 0.941 |
| CDAI <150 | 20 (80) | 19 (66) | 2.333 (0.533-10.209) | 0.261 |
| CRP <5 mg/L | 19 (76) | 18 (62) | 1.750 (0.445-6.882) | 0.423 |
| Normalisation or ≥50% reduction in CRP^‡^ | 20 (80) | 22 (76) | 0.947 (0.055-16.309) | 0.970 |
| FC <100 µg/g | 10 (40) | 13 (45) | 0.907  (0.307-2.681) | 0.859 |
| Normalisation or ≥50% reduction in FC^‡^ | 11 (44) | 19 (66) | 0.542  (0.177-1.661) | 0.284 |
| Normalisation or ≥50% reduction of CRP and FC | 10 (40) | 16 (55) | 0.571  (0.144-2.262) | 0.425 |
| FC, µg/g | 172.3 (44-1292) | 149.6 (54-855) | 107  (-263.2-477.2) | 0.564 |
| CDOS improvement, normal CRP and FC | 4 (16) | 8 (28) | 0.495 (0.18-2.081) | 0.337 |
| Endoscopy | | | | |
| Stricture passable on colonoscopy | 6/13 (46) | 5/15 (33) | 1.19  (0.265-5.348) | 0.820 |
| Stricture passable on double-balloon enteroscopy | 12/18 (67) | 6/15 (40) | 2.844  (0.918-8.81) | 0.070 |
| Improvement in SES-CD of stricture ≥1 point* | 11/13 (85) | 9/15 (60) | 1.10  (0.191-6.339) | 0.905 |
| Improvement in DBE-CD ≥1 point* | 13/18 (72) | 8/15 (53) | 3.250  (0.480-21.997) | 0.227 |
| CTE | | | | |
| CTE stricture length reduced ≥25% | 8/20 (40) | 5/18 (28) | 4.333  (0.423-44.428) | 0.217 |
| Stricture length reduced by ≥1 mm | 12/20 (60) | 11/18 (61) | 0.818  (0.149-4.505) | 0.818 |
| Stricture wall thickness improvement ≥1 mm | 9/20 (45) | 9/18 (50) | 0.583  (0.137-2.481) | 0.466 |
| Resolution of proximal dilation** | 11/20 (55) | 9/18 (50) | 1.22  (0.255-6.290) | 0.792 |
| CTE complete stricture resolution*** | 4/20 (20) | 4/18 (22) | 0.778  (0.184-3.282) | 0.732 |
| IUS | | | | |
| >25% improvement in BWT | 9 (36) | 14 (48) | 0.622  (0.211-1.836) | 0.390 |
| Normal vascularisation (Limberg score ≤1) | 8 (32) | 10(34) | 0.847  (0.270-2.653) | 0.776 |
| BWT, mm | 4.2 (3.4-5.5) | 4.6 (3.2-5.7) | 0.05  (-0.723-0.831) | 0.8901 |

CDOS=Crohn’s disease obstructive score. CDAI=Crohn’s Disease Activity Index. CRP=C-reactive protein. FC=fecal calprotectin. CTE=computed tomography enterography. IUS=intestinal ultrasound. BWT=bowel wall thickness.

† Score of 0–2 considered as symptomatic stricture remission.

‡ Normalisation in CRP (<5 mg/L) and in faecal calprotectin (<100 µg/g).

* Data reported for patients with adequate views: colonoscopy (n=28; 13 treatment-naïve, 15 previously treated) and double-balloon enteroscopy (n=33; 18 treatment-naïve, 15 previously treated). Endoscopic endpoints were only assessed in patients with a reachable or assessable stricture.

** Small bowel diameter <3.0 cm considered as resolution of proximal dilation.

*** CTE stricture resolution was defined as normal BWT and luminal diameter with absence of prestenotic dilation, and reduced stricture length by ≥1 mm.

**Supplemental Appendix 1.**

**STROBE Statement**—Checklist of items that should be included in reports of ***cohort studies***

|  | **Item No** | **Recommendation** | **Page No** |
| --- | --- | --- | --- |
| **Title and abstract** | 1 | (*a*) Indicate the study’s design with a commonly used term in the title or the abstract | 1-2 |
|  |  | (*b*) Provide in the abstract an informative and balanced summary of what was done and what was found |  |
| **Introduction** | | | |
| Background/  rationale | 2 | Explain the scientific background and rationale for the investigation being reported | 3 |
| Objectives | 3 | State specific objectives, including any prespecified hypotheses | 3-4 |
| **Methods** | | | |
| Study design | 4 | Present key elements of study design early in the paper | 15 |
| Setting | 5 | Describe the setting, locations, and relevant dates, including periods of recruitment, exposure, follow-up, and data collection | 15 |
| Participants | 6 | (*a*) Give the eligibility criteria, and the sources and methods of selection of participants. Describe methods of follow-up | 15-16 |
|  |  | (*b*) For matched studies, give matching criteria and number of exposed and unexposed |  |
| Variables | 7 | Clearly define all outcomes, exposures, predictors, potential confounders, and effect modifiers. Give diagnostic criteria, if applicable | 15-16 |
| Data sources/ measurement | 8* | For each variable of interest, give sources of data and details of methods of assessment (measurement). Describe comparability of assessment methods if there is more than one group | 16-18 |
| Bias | 9 | Describe any efforts to address potential sources of bias | 16 |
| Study size | 10 | Explain how the study size was arrived at | 15 |
| Quantitative variables | 11 | Explain how quantitative variables were handled in the analyses. If applicable, describe which groupings were chosen and why | 17-18 |
| Statistical methods | 12 | (*a*) Describe all statistical methods, including those used to control for confounding | 16-18 |
|  |  | (*b*) Describe any methods used to examine subgroups and interactions |  |
|  |  | (*c*) Explain how missing data were addressed |  |
|  |  | (*d*) If applicable, explain how loss to follow-up was addressed |  |
|  |  | (*e*) Describe any sensitivity analyses |  |
| **Results** | | | |
| Participants | 13* | (a) Report numbers of individuals at each stage of study—eg numbers potentially eligible, examined for eligibility, confirmed eligible, included in the study, completing follow-up, and analysed | 4，Fig.1A |
|  |  | (b) Give reasons for non-participation at each stage |  |
|  |  | (c) Consider use of a flow diagram |  |
| Descriptive data | 14* | (a) Give characteristics of study participants (eg demographic, clinical, social) and information on exposures and potential confounders | 4-6,  Table 1,  Table 2 |
|  |  | (b) Indicate number of participants with missing data for each variable of interest |  |
|  |  | (c) Summarise follow-up time (eg, average and total amount) |  |
| Outcome data | 15* | Report numbers of outcome events or summary measures over time | 7-11,  Fig.1B, Fig.2,  Table 3,  Table 5 |

| Main results | 16 | (*a*) Give unadjusted estimates and, if applicable, confounder-adjusted estimates and their precision (eg, 95% confidence interval). Make clear which confounders were adjusted for and why they were included | 9-10,  Fig.3,  Table 4 |
| --- | --- | --- | --- |
|  |  | (*b*) Report category boundaries when continuous variables were categorized |  |
|  |  | (*c*) If relevant, consider translating estimates of relative risk into absolute risk for a meaningful time period |  |
| Other analyses | 17 | Report other analyses done—eg analyses of subgroups and interactions, and sensitivity analyses | 9, Tables S1-S3 |
| **Discussion** | | | |
| Key results | 18 | Summarise key results with reference to study objectives | 11-13 |
| Limitations | 19 | Discuss limitations of the study, taking into account sources of potential bias or imprecision. Discuss both direction and magnitude of any potential bias | 12, 14 |
| Interpretation | 20 | Give a cautious overall interpretation of results considering objectives, limitations, multiplicity of analyses, results from similar studies, and other relevant evidence | 11-14 |
| Generalisability | 21 | Discuss the generalisability (external validity) of the study results | 14-15 |
| **Other information** | | | |
| Funding | 22 | Give the source of funding and the role of the funders for the present study and, if applicable, for the original study on which the present article is based | 19 |

*Give information separately for exposed and unexposed groups.

**Note:** An Explanation and Elaboration article discusses each checklist item and gives methodological background and published examples of transparent reporting. The STROBE checklist is best used in conjunction with this article (freely available on the Web sites of PLoS Medicine at http://www.plosmedicine.org/, Annals of Internal Medicine at http://www.annals.org/, and Epidemiology at http://www.epidem.com/). Information on the STROBE Initiative is available at <http://www.strobe-statement.org.>

**Supplemental Appendix 2. Inclusion and Exclusion Criteria**

| **Inclusion Criteria** | |
| --- | --- |
|  | Patients aged 18–80 years with a confirmed diagnosis of moderate-to-severe Crohn’s disease (CD) and treated with ustekinumab (UST). |
|  | Patients with obstructive symptoms consistent with chronic or subacute intestinal obstruction within the preceding 8 weeks. |
|  | Definite evidence of CD-related luminal stricture confirmed by computed tomography enterography (CTE) or endoscopy, meeting either criterion below:   1. CTE criteria: Intestinal stricture demonstrated on CTE, with ≥2 of the following 3 findings compared with the adjacent proximal bowel: ① >50% reduction in luminal diameter; ② >25% increase in bowel wall thickness; ③ pre-stenotic dilation >2.5 cm; 2. Endoscopic criterion: Inability to pass the endoscope across the narrowing. |
| **Exclusion Criteria** | |
|  | Severe disease requiring urgent surgery or endoscopic intervention, or medication adjustment/elective surgery indicated within 2 months by clinical assessment (e.g., acute severe intestinal obstruction, perforation, intra-abdominal abscess, adhesions). |
|  | Severe patients who failed to resume oral intake despite exclusive enteral nutrition for more than 2 months. |
|  | Previous exposure to UST or other IL-23 antagonists within the preceding 12 months, or contraindications to or intolerance of UST (e.g., previous allergic reaction to UST). |
|  | Relative contraindications to biologic agents, including active tuberculosis with positive chest radiograph or strong positive tuberculin skin test, myocardial infarction, heart failure, or demyelinating neurological disorders within the past 5 years. |
|  | Contraindications to CTE, such as contrast medium allergy. |
|  | Patients with active solid tumors, lymphoma or melanoma undergoing chemotherapy or radiotherapy. |
|  | Short bowel syndrome or severe malabsorption caused by multiple abdominal surgeries. |
|  | Complicated with active massive gastrointestinal bleeding, severe hepatic or renal dysfunction, active bacterial or viral infection, shock, etc. |
|  | Pregnant or lactating patients. |
|  | Severe hemodynamic instability, unstable vital signs, rapidly progressive disease, or end-stage disease. |
